# Spontaneous Bladder Rupture during Vaginal Delivery

**DOI:** 10.1155/criu/8756601

**Published:** 2026-01-14

**Authors:** Diogo Carmali, Júlia Quiterres, Sara Duarte, André Barcelos, Sónia Afonso Ramos, Inês Garcia Nunes, André Pita, Filipe Gaboleiro, Eduardo Felício, Guilherme Bernardo, Margarida Meira, Rute Branco, Andrea Furtado, Diogo Bruno, Ana Paula Santos, Ana Paula Ferreira, Fernando Ferrito

**Affiliations:** ^1^ Urology Department, Unidade Local de Saúde Amadora Sintra, Amadora, Portugal; ^2^ Obstetrics and Gynecology Department, Unidade Local de Saúde Amadora Sintra, Amadora, Portugal

## Abstract

Spontaneous bladder rupture during vaginal delivery is a rare yet serious complication that requires prompt recognition and management to prevent life‐threatening consequences. We report the case of a 32‐year‐old woman with a previous cesarean section who developed concurrent uterine and bladder rupture following vaginal delivery after labor induction, a highly unusual occurrence. The presence of hematuria right before delivery, which worsened postpartum, raised suspicion for a bladder injury. Diagnosis was confirmed intraoperatively through laparotomy. Timely surgical repair was performed, leading to a favorable outcome and full recovery.

## 1. Introduction

The close anatomical relationship between the lower urinary and reproductive tracts predisposes the bladder to iatrogenic injury during obstetric procedures [1]. The bladder trigone rests on the anterior vaginal fornix, while its base is in proximity to the lower uterine segment and cervix, making it particularly vulnerable during obstetric interventions. Spontaenous bladder rupture during labor or postpartum is an uncommon but severe event, typically caused by prolonged fetal head compression and excessive uterine contractions, which may induce pressure necrosis of the bladder dome [2]. Factors such as increased visceral pressure, bladder wall weakening, and vesical catheterization during labor contribute to its development [3]. Clinically, patients may present with suprapubic pain, anuria, or hematuria, making early diagnosis challenging [2]. Due to its severity, bladder rupture constitutes a surgical emergency, requiring prompt recognition and management to prevent life‐threatening complications [4, 5]. This case report was written in accordance with the CARE guidelines and follows the recommendations of the International Committee of Medical Journal Editors (ICJME) for authorship and publication ethics. Written informed consent was obtained from the patient for publication of this case report and any accompanying images.

## 2. Case Report

A 32‐year‐old Caucasian woman (height 1.63 m and weight 72 kg), Gravida 2 Para 1, at 41 + 0 weeks of gestation, was admitted for labor induction due to advanced gestational age, according to the hospital protocol. Her obstetric history included a cesarean section 4 years before (the indication for which is unclear/unknown) but no other surgeries or significant medical conditions.

Labor induction was initiated with a Foley catheter, followed the next day by a vaginal dinoprostone insert.

A few hours later, cardiotocography detected uterine tachysystole, which was solved with intravenous salbutamol. Epidural analgesia was administered. Oxytocin was not administered. Then, 5 h after dinoprostone insertion, spontaneous rupture of membranes occurred, with clear amniotic fluid, and 6 h after administration, the cervix was fully dilated. However, 9 h after dinoprostone administration, due to a nonreassuring fetal status, an assisted vaginal delivery with forceps was performed.

A healthy newborn weighing 3060 g was delivered, with Apgar scores of 9/10/10. Right before delivery, bladder catheterization revealed hematuria, which was not initially considered significant, as it was assumed to be of traumatic origin related to the catheterization. However, the hematuria worsened in the immediate postpartum period. Ultrasound examination showed a bladder and uterus containing heterogeneous content consistent with blood clots, as well as free fluid in the periuterine space. Suspecting uterine rupture with possible extension to adjacent structures, an exploratory laparotomy was performed approximately 1–2 h after delivery.

### 2.1. Intraoperative Findings and Surgical Management

**Figure 1:**
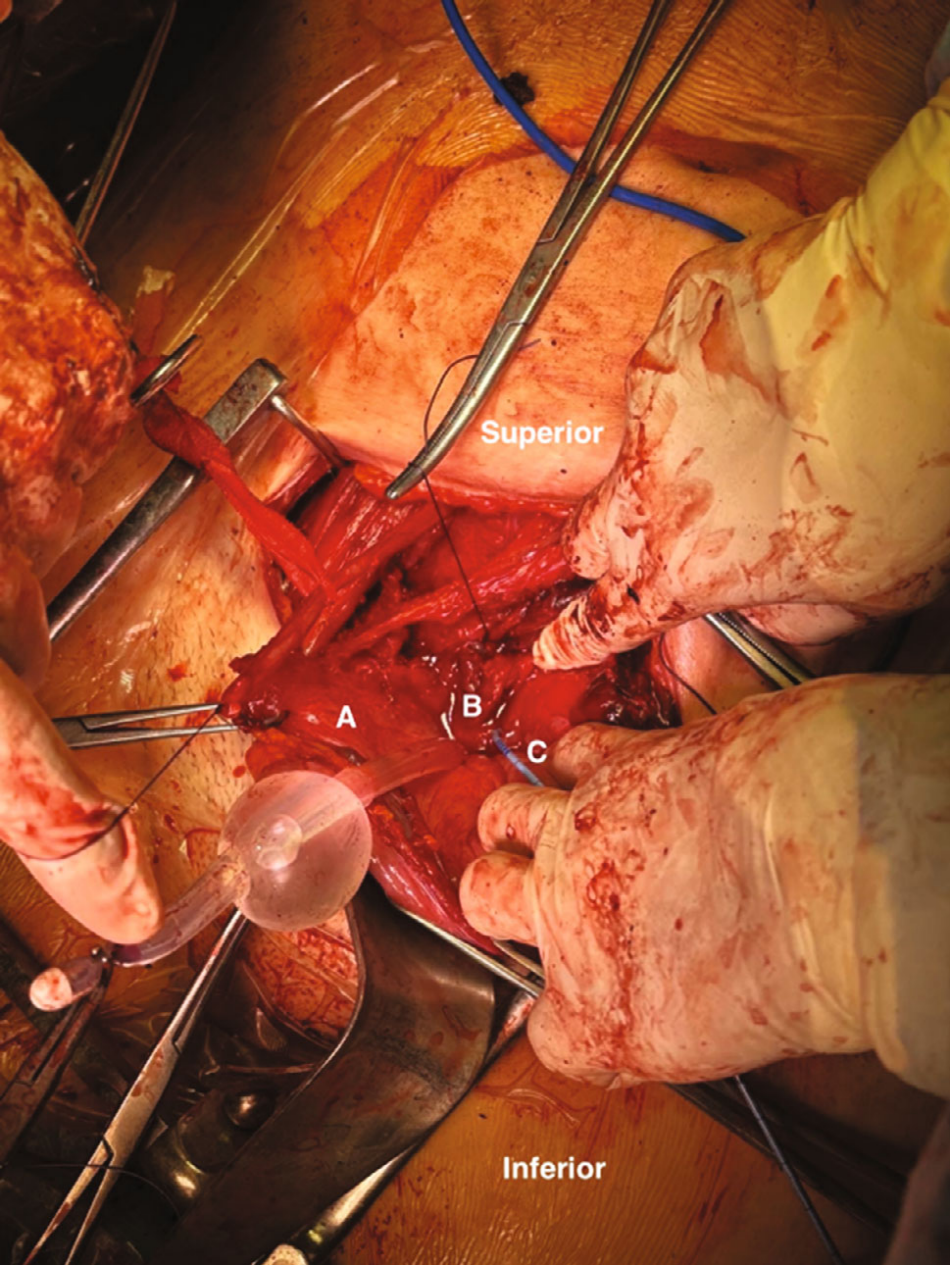
Intraoperative view. (A) Bladder anterior wall. (B) Bladder trigone. (C) Left ureteral catheter.

**Figure 2:**
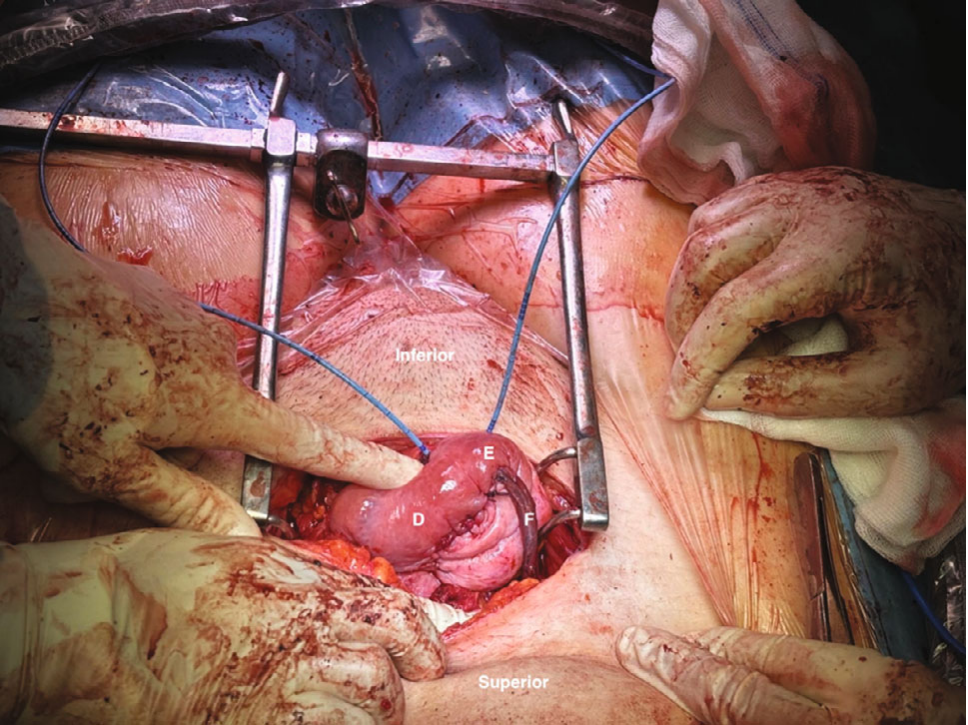
Intraoperative view. (D) Bladder posterior wall. (E) Bladder dome. (F) Reimplanted right ureter.

Intraoperatively, a uterine rupture was identified, with a full‐thickness laceration of the anterior uterine wall extending from the previous cesarean scar down to the cervix. The rupture was initially contained by the uterine serosa, with a small amount of free hemoperitoneum. Upon opening the serosa, profuse hemorrhage (~1000 mL) was encountered. A full‐thickness complex bladder rupture was identified, radiating in a fan‐like pattern from the bladder dome to the trigone area, involving the anterior, fundal, and posterior walls. The anterior uterine segment was repaired with multilayer sutures, which were laborious due to the absence of well‐defined tissue planes between the uterus and bladder. After achieving hemostasis, the bladder walls were dissected laterally and anteriorly. The left ureter was catheterized with a Terumo guidewire, confirming patency, and a Mono‐J catheter was placed up to the kidney, as shown in Figure 1. A laceration at the right ureteral meatus was identified, preventing its retrograde catheterization. Thus, the decision to reimplant the ureter was made. After retroperitoneal dissection, the right ureter was ligated at the pelvic level. Then, a Terumo guidewire was advanced to the right kidney, and a Mono‐J catheter was placed. The right ureter was reimplanted into the neobladder dome using a Lich–Gregoir anastomosis, as shown in Figure 2. Afterwards, the bladder defect was repaired in a star‐shaped suture configuration using 2‐0 Vicryl, successfully restoring its anatomical contour. A 20Ch silicone Foley catheter was inserted, and the Mono‐J catheters were exteriorized through the anterior bladder wall. A methylene blue leak test confirmed bladder integrity, with no leak detected. A silastic drain was placed in the right pubic region, and the ureteral catheters were exteriorized through the left pubic region. The abdominal wall was closed in layers with an intradermal suture on the skin. During surgery, the patient received 1 g of tranexamic acid and two units of packed red blood cells.

### 2.2. Postoperative Course

**Figure 3:**
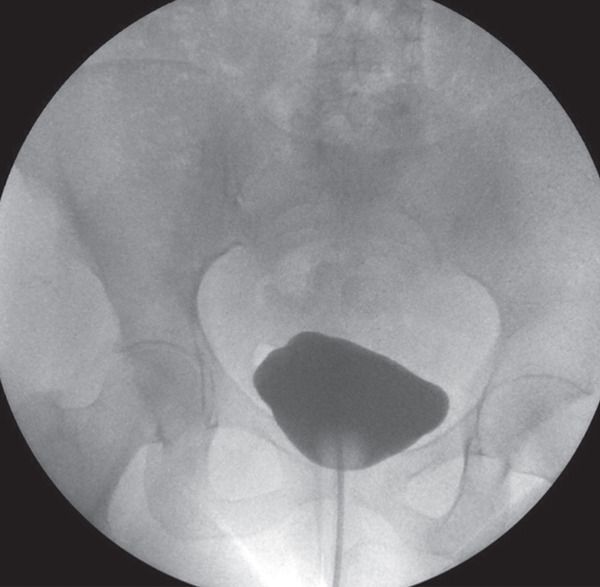
Retrograde cystography at three‐week follow‐up showing no evidence of extravasation.

Postoperatively, the patient remained clinically stable and received intravenous ceftriaxone daily (2 g), according to hospital protocol for prophylaxis in complex genitourinary surgery. Over the following days, hematuria from the ureteral catheters gradually cleared, inflammatory seric markers improved and both the bladder catheter and silastic drain maintained only residual output. By the fifth postoperative day, urine was clear through ureteral catheters and by Day 7, drainage from the silastic drain was negligible, allowing its removal. The patient was discharged on the seventh postoperative day, with instructions to maintain high fluid intake to ensure adequate urinary output and facilitate healing, and medicated with prophylactic cefuroxime, based on local guidelines for extended perioperative coverage. At 1‐week follow‐up, she was asymptomatic, with normal and clear diuresis both from ureteral and bladder catheters. By then, ureteral Mono‐J catheters were removed. At 4‐week follow‐up, the patient underwent retrograde cystography, which showed no evidence of extravasation, as shown in Figure 3. Based on these findings, the bladder catheter was removed. From then on, the patient remained asymptomatic with no clinically significant voiding dysfunction as confirmed by uroflowmetry (Qmax 17.7 mL/s, voided volume 184.4 mL, and postvoid residual 90 mL).

## 3. Discussion

Spontaneous bladder rupture following vaginal delivery is exceedingly rare but can have life‐threatening consequences if not promptly diagnosed and managed. Due to the limited data available in the literature, its physiopathology remains poorly understood, frequently leading to a delayed diagnosis. Nevertheless, it is often linked to factors such as recent trauma, prior instrumentation, anatomical obstruction, malignancy, prolonged catheterization, or neurogenic bladder dysfunction [6].

Although diagnosis and management were timely, this case illustrates several risk factors for uterine and bladder rupture: such asprior cesarean section, and bladder catheterization. Awareness of these factors may prompt earlier recognition of hematuria or abdominal signs, potentially preventing delayed diagnosis. Careful monitoring, judicious use of induction agents, and adherence to labor guidelines are crucial for minimizing such rare complications.

According to the guidelines of the Portuguese Society of Obstetrics and Maternal‐Fetal Medicine (SPOMMF), both controlled release vaginal dinoprostone inserts and mechanical methods are considered safe options for labor induction in women with a previous cesarean section who are candidates for a trial of labor, provided specific clinical criteria are met [7]. Randomized controlled trials have demonstrated that the combined use of Foley catheter and a vaginal dinoprostone insert is both feasible and effective for cervical ripening and labor induction. Studies show that simultaneous administration of both methods can reduce the induction‐to‐delivery interval and may lower the cesarean delivery rate compared to Foley catheter alone [8, 9].

Clinical signs typically include abdominal pain, abdominal tenderness and anuria [10]. Some cases have also reported acute renal failure, which is reflected in laboratory findings, including elevated levels of creatinine, urea, and potassium, as well as decreased sodium and chloride concentrations [11–13]. Dysuria and hematuria are less frequently observed, which may lead to misdiagnosis or diagnostic delays, as these symptoms could divert suspicion from a urological etiology [14]. Postpartum urinary retention is a major risk factor, with epidural anesthesia increasing its likelihood threefold. Other recognized risk factors include the use of systemic narcotics, perineal lacerations, instrumental delivery, and an overdistended bladder during labor, particularly in the absence of catheterization [14, 15]. In addition, inadequate catheterization during labor has also been identified as a contributing factor in several studies, particularly when performed hastily with excessively thin catheters or excessive force. [16] Imaging plays a crucial role in the diagnosis of bladder rupture. Ascending cystography remains the gold standard, allowing direct visualization of bladder integrity and contrast leakage [2]. However, not all centers, including ours, are capable of performing cystography in an emergency setting outside the operating room, which may lead to delayed diagnosis and intervention. In such cases, ultrasound can be a valuable alternative. Although it cannot directly visualize bladder rupture, ultrasound can detect free fluid in the peritoneal cavity, which may suggest a potential rupture or other abdominal issues. Ultrasound is noninvasive, widely available, and can guide further diagnostic and therapeutic decisions, making it an essential tool in settings where more advanced imaging techniques may not be immediately accessible. Management of bladder rupture depends on whether the rupture is intra‐ or retroperitoneal. Early diagnosis and prompt surgical intervention are crucial for reducing morbidity and mortality. Extraperitoneal ruptures are typically managed conservatively with bladder catheterization for approximately 10 days. In contrast, intraperitoneal ruptures usually require surgical repair, which involves drainage of the urine accumulated in the peritoneal cavity, closure of the rupture, and ensuring adequate bladder emptying [3, 13, 17, 18]. Traditionally, laparotomy has been the standard surgical approach, but laparoscopy can be a viable alternative in hemodynamically stable patients, particularly when performed by experienced surgeons [2].

It is important to note that reporting such cases is valuable for increasing awareness of proper selection of candidates for a trial of labor after cesarean section. Despite not being exceptionally rare, due to adhesions between the bladder and lower uterine segment after previous cesarean section, uterine rupture can involve the bladder as seen in this patient.

Our case underscores the importance of considering bladder rupture in postpartum patients with unexplained hematuria, especially those with a history of prior cesarean section and assisted delivery.

## 4. Conclusion

In conclusion, spontaneous bladder rupture though uncommon, remains a life‐threatening emergency that requires prompt diagnosis and intervention. Despite its low incidence and nonspecific symptoms, the condition is often associated with factors such as postpartum urinary retention, improper catheterization, and other contributing obstetric risks. Diagnostic challenges arise due to the lack of immediate access to advanced imaging in some settings, making ultrasound a valuable alternative when more sophisticated tools are unavailable. Early and accurate diagnosis is critical for reducing morbidity and mortality, and surgical management, typically through laparotomy or laparoscopy, remains the cornerstone of treatment. Ultimately, recognizing risk factors, employing appropriate diagnostic approaches, and selecting the optimal surgical technique are key to improving outcomes in these rare but serious cases. This case highlights the importance of early recognition and surgical management of bladder rupture during assisted vaginal delivery, especially in patients with a history of previous cesarean section.

## Ethics Statement

This study was exempt from ethical review given its nature as a case report.

## Consent

All authors and patients have provided consent for publication.

## Conflicts of Interest

The authors declare no conflicts of interest.

## Author Contributions

**Diogo Carmali:** writing – original draft, supervision, resources, methodology, conceptualization. **Júlia Quiterres**: writing – original draft, supervision, resources, methodology, conceptualization. **Sara Duarte, Inês Garcia Nunes, André Pita, Filipe Gaboleiro, Eduardo Felício, Guilherme Bernardo, and Margarida Meira:** investigation, data curation, software, resources, methodology. **André Barcelos, Sónia Afonso Ramos and Rute Branco**: writing – review and editing, formal analysis, supervision, conceptualization. **Andrea Furtado, Diogo Bruno, and Ana Paula Santos:** conceptualization, formal analysis**. Ana Paula Ferreira and Fernando Ferrito**: supervision, validation, project administration. **Diogo Carmali and Júlia Quiterres** contributed equally to this case report and wish to be considered first authors.

## Funding

No funding was received for this manuscript.

## Data Availability

The corresponding author is at your disposal for any further data information.
